# Longevity of the Friends

**Published:** 1881-02

**Authors:** 


### LONGEVITY OF THE FRIENDS.

On opening a weekly periodical which comes among our exchanges, called "*The Journal*," published in Philadelphia, in the interest of the Society of Friends, we notice the record of eight deaths, the ages being as follows: 73, 93, 78, 78, 89, 86, 16 and 80. The average age of the seven, omitting the death at 16, was upwards of 82 years. The friends are, almost to a unit, abstainers from intoxicating drinks.

There is also a biographical sketch of Samuel M. Janney, late of Loudon County, Virginia, an eminent minister in the same religious body, who died some months ago, at the age of 78 years. Though not coming from a medical source, it mentions an important fact in connection with his history, illustrative of the proper treatment of incipient or threatened pulmonary consumption. At the age of 25, symptoms of that disease appearing, "he was induced to travel, and to seek in the healing waters of Red Sulphur Springs, in Virginia, a remedy for his enfeebled condition." He visited those springs occasionally, and attributed his recovery to these journeys, and to the frequent rides on horseback taken for a number of years. This was more than fifty years ago, and nothing has been discovered since that time more effectual in averting death under similar circumstances. The horse and saddle, not the water, did the good work.

In the *Comptes Rendus*, No. 17, M. J. Salleran says he often meets with well made thermometers, the indications of which are erroneous to 8° or 10°, or even more. Such changes occur at printing ink works, where oils are heated for several days to 270°; in glycerine works, and with rectifiers of benzol. Glass is not merely modified when heated to 300°; it undergoes true deformation at far lower temperatures. Thus, the hydrometers used in sugar works, which are often exposed for a considerable time at temperatures of 95°, are affected. After an immersion of some days they are completely modified, their weight decreases, and they become erroneous to the extent of 7° to 8° B. The editor of the Chemical News adds that in many chemical works it has been found necessary to submit all hydrometers used for hot liquids to a weekly comparison with a standard instrument.

